# Independent and opposing effects of self-complexity and weight-based social identity threat on well-being among larger-bodied people

**DOI:** 10.3389/fpsyt.2026.1810126

**Published:** 2026-06-10

**Authors:** Lukas Loreth, Julian Paffrath

**Affiliations:** University of Kiel, Kiel, Germany

**Keywords:** self-complexity, stigmatization, weight stigma, weight-based social identity threat, well-being

## Abstract

This article examines the antagonistic effects of self-complexity and weight-based social identity threat on well-being among larger-bodied people. To this end, we conducted two experiments in which we manipulated self-complexity using a trait sorting task adapted for online studies and manipulated the salience of weight-based social identity threat using the mental imagery task. The results of both studies provided limited evidence that high self-complexity counteracts the negative effects of weight-based social identity threat. While the exploratory findings of Study 1 (*N* = 405) suggested that having a high positive self-complexity could counteract or buffer the effects of weight-based social identity threat, Study 2 (*N* = 335) did not replicate those findings. These results are discussed in terms of the theoretical implications of self-complexity effectiveness, the self-complexity manipulation employed, and differences in sample demographics.

## Introduction

Weight stigma refers to societal devaluation of people due to their body weight or body size and is a widespread phenomenon (1, 2). Larger-bodied people are often reduced to their body weight as the only relevant aspect of their self, which becomes the basis for how they are perceived, described, and treated in social contexts. Common stereotypes depict them as lazy, lacking willpower, or undisciplined (3), and they face discrimination across multiple domains, including employment (4, 5), healthcare (6, 7), and education (8, 9). Numerous studies show that weight stigma is associated with a range of adverse outcomes, including disadvantages in hiring, wages, promotions, and job termination, increased distress, reduced well-being, impaired social functioning, and poorer psychological and physical health (e.g., 2, 10). Perceived weight stigma increases vulnerability to experiencing weight-based social identity threat, a phenomenon wherein people are concerned about being stigmatized solely due to their body weight (1, 11). This threat can affect individuals’ sense of belonging, feelings of loneliness, self-worth, as well as psychological and physical health (e.g., 1, 2, 12).

Although there is a rich body of research demonstrating the negative impact weight-based social identity threat has on individuals’ psychological and physical health (see 2), less is known about potential protective factors that may counteract its impact. The present work aims to address this gap by investigating whether self-complexity serves as a psychological resource that antagonizes the negative effects of weight-based social identity threat. To this end, we conducted two online experiments with larger-bodied people living in Germany as participants.

## Theoretical background

### Weight-based social identity threat and its consequences on health outcomes

Larger-bodied people experience weight-based social identity threat in social contexts in which situational cues trigger a concern that they might be stigmatized because of their body weight (1, 11). This threat can arise from the mere awareness that one belongs to a culturally devalued group, combined with situational cues that reinforce this devaluation (11, 13). As larger-bodied people are highly aware of the culturally shared weight stigma, they are highly vulnerable to experiencing social identity threat, triggered by both implicit and explicit cues (1, 2, 10). For example, implicit social identity threat may occur through disapproving looks while eating in public or exposure to media depicting larger-bodied people as lazy or lacking self-control (1). Explicit social identity threat may occur through ‘fat jokes,’ insults, or derogatory comments (1). When such experiences accumulate, they may lead to chronic social identity threat (11, 14).

Based on the stigma-induced social identity threat model, these experiences impair physical and psychological health by elevating stress and negative emotions (1, 2). Those affected often perceive such situations as overwhelming and difficult to cope with (2, 13), which can elicit negative emotions, such as anger, fear, and/or shame (e.g., 13, 15, 16). As correlational and experimental research has shown, this can lead to inflammatory responses (17), greater cardiovascular reactivity (18, 19), elevated ambulatory blood pressure (20), increased activation of the hypothalamic-pituitary-adrenal axis and cortisol release (21), lower self-esteem, poorer quality of life, greater depression and anxiety disorders (1, 22, 23), as well as higher body image dissatisfaction (24). Moreover, social identity threat undermines individuals’ sense of social belonging, increasing feelings of loneliness (13, 25).

### Self-complexity and its consequences on health outcomes

Self-complexity pertains to how individuals cognitively structure their self-concept. Linville (26, 27) defined self-complexity as the number of independent self-aspects to organize one’s self-knowledge. Individuals differ in their number of self-aspects and the amount of independence—i.e., (non-)overlap—between them. Self-aspects can include individual characteristics or group affiliations and associated group characteristics, representing the individual or collective self (26–29). For example, individuals might think of themselves in terms of their physical characteristics (e.g., body image), abilities (e.g., being talented), social roles (e.g., being a grandparent), group memberships or categories (e.g., being a member of a book club), and so forth. The amount of independence between self-aspects is determined by the level of distinct attributes (e.g., caring or intelligent) that are ascribed to each self-aspect. High self-complexity is characterized by numerous and non-overlapping self-aspects, whereas low self-complexity involves fewer or highly overlapping self-aspects.

According to the self-complexity model, individuals with higher self-complexity are likely to experience higher well-being than individuals with lower self-complexity (26, 27). When confronted with a stressor, threatening well-being, it negatively affects the self-aspect that is most relevant to the situation. For individuals high in self-complexity, the negative impact is limited because only a small proportion of their self-concept is affected. In contrast, for individuals low in self-complexity a greater proportion of their self-concept is negatively affected. Furthermore, low self-complexity facilitates a spillover effect, whereby the stressor affecting one self-aspect also influences closely linked self-aspects (26, 27). Empirical support for the stress-buffering role of self-complexity stems from studies examining both individual and collective self-complexity. Initial support for the individual self-complexity approach stems from Linville (27), who used a trait-sorting task to assess individual self-complexity and found that it moderated the relationship between stress and health. Specifically, participants with high individual self-complexity reported fewer physical and psychological symptoms following high-stress experiences.

Support for the collective self-complexity approach stems from the social cure literature, which demonstrated that group memberships can provide important psychological resources, such as social support and a sense of belonging, which are associated with improved well-being (30–33). However, this work has typically relied on measures that capture isolated aspects of social identity (e.g., number of group memberships), thereby providing only a limited view of individuals’ broader social identity structure. To address this limitation, Bentley et al. (30) developed the Social Identity Mapping (SIM) tool, designed to capture the complexity of individuals’ social identity structures by assessing not only the number of group memberships, but also their qualitative characteristics and interrelations. This tool was found effective to help individuals recognize important and meaningful group memberships, that provide them with psychological resources (e.g., meaning, belonging, purpose, and control) and thereby improving their overall wellbeing (30, 32, 33).

Banas and Smyth (28) developed and tested a hybrid approach that mixes the individual and collective self-complexity approaches in a preregistered correlational study. More specifically, the authors adapted Shower’s self-descriptive card sorting task for online research (34, 35). Participants were first asked to list as many self-aspects of themselves as they wanted and then selected as many attributes as they felt necessary to describe these self-aspects from a list of 20 positive and 20 negative attributes (28). The results supported the stress-buffering effect of high individual and high collective self-complexity on well-being. Specifically, analyses of very positive self-aspects—i.e., superaspects—showed that, after controlling for stress, a greater number of individual superaspects was associated with higher affective well-being, whereas a greater number of collective superaspects was associated with both higher affective well-being and quality of life. Further analyses indicated that these associations were not limited to superaspects, though the relationships were comparatively weaker. However, the authors suggested that future studies should further explore the potential distinct benefits of individual and collective self-aspects.

Taken together, previous theoretical reflections and empirical research suggest that the beneficial effects of self-complexity may depend on the type of self-aspect (individual vs. collective) as well as on the positivity of self-aspects under conditions of stress. Following the suggestion of Banas and Smyth (28), the present research strives to experimentally disentangle these components by examining the distinct effects of individual and collective self-complexity (Study 1) and the role of positivity for individual self-complexity (Study 2) in buffering weight-based social identity threat.

## The present research

It remains an open question whether *stigmatized* individuals benefit similarly from high self-complexity, particularly when experiencing implicit or explicit social identity threat, and whether individual and collective forms of self-complexity differentially affect their well-being. The present research addresses this gap by examining the antagonistic main effects of weight-based social identity threat and self-complexity on well-being, testing whether higher social identity threat is associated with lower well-being and whether higher self-complexity is independently associated with greater well-being. Furthermore, we investigate their interaction to determine whether higher self-complexity buffers the negative impact of social identity threat, such that individuals with higher self-complexity experience smaller declines in well-being following threatful situations.

## Study 1

In Study 1, we expected antagonistic main effects of both independent variables: We hypothesized high individual and high collective self-complexity, compared to low self-complexity, to show positive main effects on sense of social belonging, self-esteem, and body satisfaction as well as negative main effects on negative affect, loneliness, and depressive symptoms. While high individual and high collective self-complexity were expected to have equivalent effects, stronger effects of high collective self-complexity on social belonging and loneliness could be expected based on the social cure literature (e.g., 30, 32, 33). We hypothesized weight-based social identity threat—with the levels no, implicit, and explicit—to show a negative main effect on the former dependent variables as well as a positive main effect on the latter dependent variables. We also expected an interaction effect of both independent variables: We hypothesized self-complexity to buffer the negative effects of weight-based social identity threat on all dependent measures. Again, the buffering effect of high collective self-complexity as opposed to high individual self-complexity could be expected to be stronger for sense of social belonging and loneliness. In all other cases, high individual and high collective self-complexity were expected to have equivalent effects.

### Method

#### Participants

An a-priori power analysis for a two-way general linear model (GLM) with nine conditions and *df* = 4 using G*Power (36) revealed that we needed to collect a minimum of *N* = 396 to detect a small to medium effect of *f* = 0.175, assuming an *α* = .05, and *1-β* = .80. Participants were recruited via an online sampling provider (*Bilendi GmbH*; www.bilendi.com). The present study was introduced to participants as an investigation of how one is perceived by others in social interactions. To account for potential drop-out, we decided to oversample by approximately 10%. In total, 431 larger-bodied people living in Germany completed the study. Twenty-six participants were excluded because they did not follow instructions of the self-complexity task by either entering the same self-aspect multiple times or by responding with nonwords. This resulted in a total sample size of 405 participants. Based on self-reported BMI, 55 participants (13.58%) had a BMI between 25.0 and 29.9, and 350 participants (86.42%) had a BMI of 30.0 or higher (no missing values). The average age of the sample was *M* = 52.10 years (*SD* = 13.30). One hundred and ninety-five (48.15%) participants were male, 209 (51.60%) participants were female, and one (0.25%) participant reported to be non-binary (no missing values). Nine (2.22%) participants reported having a migration background (no missing values), 97 (23.95%) participants had a college or university degree (two missing values), and five (1.23%) participants reported to be a supporter of the body positivity movement (16 missing values).

#### Design and procedure

We conducted an online experiment using a 3x3 between-subjects design manipulating self-complexity (low vs. high individual vs. high collective) and the salience of social identity threat (no vs. implicit vs. explicit). Participants were invited through an online sampling provider (*Bilendi GmbH*; www.bilendi.com). After clicking on the survey link, participants were redirected to the welcome page of our survey, followed by a pre-screening question, in which they had to self-categorize themselves as a larger-bodied person, as well as indicate their BMI. Eligibility required participants to self-categorize as larger-bodied and report a BMI of at least 25; those who did not meet both criteria were excluded and redirected to the sampling provider.^1^ Eligible participants provided informed consent and were then randomly assigned to one of the three self-complexity conditions.

To manipulate self-complexity, we adapted the procedure from Banas and Smyth (28). In the high individual self-complexity condition, participants were instructed to list at least two self-aspects that are important to them and describe them in a very personal and individual way. Below the instructions participants were presented with several free text lines to type in their responses. In the top line participants were presented with the self-aspect “I am a larger-bodied person.” In the high collective self-complexity condition, participants were instructed to list at least two additional self-aspects that were important to them, that they share with others, and describe them as being part of a group. In the top line of the free response field, participants were presented with the self-aspect “Us larger-bodied people.” Next, participants were instructed to ascribe at least two attributes from a list of 50 positive (e.g., accepting, diligent, or tidy) and 50 negative attributes (e.g., aimless, bored, or unimaginative) to each self-aspect they have listed (28, 38). Each attribute could be used as many times as desired. In the control low self-complexity condition, participants were not instructed to list any self-aspects. Instead, they were instructed to ascribe at least two attributes to their self-aspect “I am a larger-bodied person.

The social identity threat manipulation followed, which was adapted from Loreth and Simon (25). Participants were randomly assigned to one of three threat conditions. In all three conditions, participants were instructed to visualize a scenario as vividly as possible and to fully immerse themselves in it. The scenario was consistent across groups but varied in the level of social identity threat induced. Participants were asked to imagine themselves at a house party in their neighborhood, seated at a table near other guests. In the no threat control condition, participants visualized the setting without any threat cues. They visualized sitting at a table while people at a neighboring table talked, with one person briefly glancing in their direction. In the implicit threat condition, they visualized people talking at a neighboring table, with one person briefly gazing at them while they ate. In the explicit threat condition, the scenario was the same, but one person at the neighboring table made a clearly negative comment about their eating behavior.

After participants read the manipulation text, they filled out several scales in the following order: measured social identity threat (manipulation check), affective responses (anger, fear, and shame), sense of social belonging, loneliness, self-esteem, body satisfaction, and depressive symptoms. Consistent with the context-dependent nature of social identity threat, all dependent variables were assessed as state measures to reflect participants’ momentary responses. Then, participants indicated the level of positivity for each of their self-aspects. Next, participants answered socio-demographic questions about their age, gender, level of education, migration background, and being a supporter of the body positivity movement. Finally, participants were debriefed, given the opportunity to give feedback, thanked for participation, and redirected to the online survey provider to be paid for their participation.

#### Materials

##### Self-complexity indices

To check whether our experimental manipulation of self-complexity was successful, we calculated the commonly used *H* dimensionality statistic (27) using a SPSS macro (39, 40). The calculation of *H* is shown in Equation 1:

(1) $$H=\text{ lo}{\text{g}}_{2}n-\left(\sum n_{i}\text{lo}{\text{g}}_{2}n_{i}\right)/n.$$

Here, *n* is the total number of attributes supplied (in this case 100), *n*_i_ is the number of attributes in the *i^th^* group combination, *i* = 1, …, 7^k^ with *k* as the number of self-aspects groups, and $n=\;\sum n_{i}$. A higher *H*-score indicates a higher self-complexity, whereas a lower *H*-score indicates a lower self-complexity (27).

##### Positivity of each self-aspect

The level of positivity of each self-aspect was measured using a single item (28, 30). Participants rated each self-aspect on a 10-point scale ranging from 0 (not at all positive) to 10 (very positive). This measure was used to compare the overall valence of the self-aspects across groups, check for potential spillover effects of the stigmatized self-aspect on other self-aspects, and to calculate the number of superaspects for the exploratory analyses.

##### Social identity threat

To check whether our experimental manipulation of social identity threat was successful, we adapted the twelve items from Loreth and Simon (25; e.g., “In this situation, I worry that people judge me because I am larger-bodied”). Participants responded by indicating their level of agreement on a 7-point scale ranging from 0 (disagree completely) to 6 (agree completely). The scale showed very good internal consistency (*α* = .98).

##### Affective responses

Participants’ affective responses to the specific experimental condition were assessed with 13 emotion words (25). Participants responded by indicating the extent to which they felt each emotion on a 7-point scale ranging from 0 (not at all) to 6 (extremely). Anger was measured as the composite of anger, fury, rage, and irritation. Fear was measured as the composite of fear, anxiety, worry, concern, and nervousness. Shame was measured as the composite of shame, embarrassment, humiliation, and self-consciousness. The anger composite (*α* = .94), fear composite (*α* = .95), and shame composite (*α* = .93) showed very good internal consistencies.

##### Sense of social belonging

Sense of social belonging was measured using the four items from Loreth and Simon (25; e.g., “In this situation, I feel accepted”). Participants responded by indicating the level of their agreement on a 7-point scale ranging from 0 (disagree completely) to 6 (agree completely). The scale showed very good internal consistency (*α* = .95).

##### Loneliness

Loneliness was measured using the five items with the highest factor loadings of the German version of the R-UCLA Loneliness Scale (41; e.g., “In this situation, I feel completely alone”). Participants responded by indicating the level of their agreement on a 7-point scale ranging from 0 (disagree completely) to 6 (agree completely). The scale showed very good internal consistency (*α* = .96).

##### Self-esteem

Self-esteem was measured with the ten item Rosenberg self-esteem scale (42; e.g., “I take a positive attitude toward myself”). Participants responded by indicating the level of their agreement on a 4-point scale ranging from 0 (disagree completely) to 3 (agree completely). The scale showed very good internal consistency (*α* = .93).

##### Body satisfaction

Body satisfaction was measured using three items adapted from Tylka and Wood-Barcalow (43; e.g., “In this situation, I feel comfortable in my body”) and supplemented by two additional items (e.g., “In this situation, I am satisfied with my body”). Participants responded by indicating the level of their agreement on a 7-point scale ranging from 0 (disagree completely) to 6 (agree completely). An exploratory factor analysis using varimax rotation indicated that all items loaded on a single factor, explaining 90.47% of the variance. The scale showed very good internal consistency (α = .97).

##### Depressive symptoms

Depressive symptoms were measured using the seven items of the depression subscale from the German Depression, Anxiety and Stress Scale 21 (DASS-21; 44; e.g., “In this situation, I felt that life was meaningless”). Participants responses were captured on a 4-point scale ranging from 0 (does not apply to me at all) to 3 (applies to me very much). The scale showed very good internal consistency (*α* = .95).

### Results

All analyses reported in this article were carried out using *IBM SPSS Statistics 29* (45). For ANOVAs reported in the text, we present only significant effects and the corresponding *post hoc* comparisons. Full results for all ANOVAs are provided in **Tables 1**–**3**. The subsequent preliminary analyses and hypothesis tests were conducted using 3x3 factorial ANOVAs.

**Table 1:** Means, standard deviations, and analyses for the interaction of self-complexity and social identity threat on study variables (Study 1).

Variable	Low SC			High individual SC			High collective SC			ANOVA				
	*M*	*SD*	*n*	*M*	*SD*	*n*	*M*	*SD*	*n*	Effect	*F* ratio	*df*	*p*	$\eta_{p}^{2}$
Positivity of self-aspects														
No Threat	2.29	2.69	35	6.98	1.37	33	6.75	2.15	37	SC	174.17	2, 392	<.001	.47
Implicit Threat	2.89	2.44	56	6.93	2.02	47	6.79	2.05	37	Threat	0.24	2, 392	.789	.00
Explicit Threat	2.55	2.68	60	7.00	1.67	58	6.82	2.07	38	SC x Threat	0.30	4, 392	.877	.00
*H* (MC)														
No Threat	0.22	0.08	36	0.53	0.21	33	0.57	0.22	37	SC	147.78	2, 396	<.001	.43
Implicit Threat	0.21	0.08	57	0.64	0.34	48	0.55	0.21	38	Threat	1.23	2, 396	.293	.01
Explicit Threat	0.23	0.08	60	0.55	0.21	58	0.52	0.14	38	SC x Threat	1.72	4, 396	.146	.02
Social identity threat (MC)														
No Threat	1.94	1.74	36	1.44	1.56	33	2.42	1.93	37	SC	2.68	2, 396	.070	.01
Implicit Threat	2.33	1.66	57	1.88	1.67	48	1.56	1.68	38	Threat	15.95	2, 396	<.001	.07
Explicit Threat	3.11	1.88	60	2.65	1.86	58	3.15	1.91	38	SC x Threat	1.73	4, 396	.143	.02
Anger														
No Threat	0.76	1.22	36	0.59	0.92	33	0.95	1.32	37	SC	0.57	2, 395	.565	.00
Implicit Threat	1.14	1.41	57	1.10	1.54	47	0.62	1.22	38	Threat	44.85	2, 395	<.001	.19
Explicit Threat	2.48	1.72	60	2.18	1.64	58	2.31	1.82	38	SC x Threat	1.02	4, 395	.398	.01
Fear														
No Threat	1.51	1.72	36	0.97	1.06	33	1.53	1.76	37	SC	1.60	2, 395	.204	.01
Implicit Threat	1.34	1.55	57	1.37	1.61	47	0.91	1.36	38	Threat	2.46	2, 395	.087	.01
Explicit Threat	1.84	1.60	60	1.38	1.47	58	1.58	1.54	38	SC x Threat	1.18	4, 395	.318	.01
Shame														
No Threat	1.72	1.86	36	1.18	1.31	33	1.77	1.80	37	SC	1.95	2, 395	.144	.01
Implicit Threat	1.70	1.57	57	1.45	1.63	47	1.09	1.57	38	Threat	12.66	2, 395	<.001	.06
Explicit Threat	2.60	1.87	60	2.19	1.95	58	2.33	1.78	38	SC x Threat	0.76	4, 395	.552	.01
Sense of social belonging														
No Threat	2.66	1.66	36	2.70	1.45	33	2.07	1.29	37	SC	0.80	2, 395	.452	.00
Implicit Threat	2.54	1.63	56	2.61	1.44	48	2.73	1.35	38	Threat	29.55	2, 395	<.001	.13
Explicit Threat	1.46	1.49	60	1.45	1.40	58	1.29	1.40	38	SC x Threat	0.89	4, 395	.470	.01
Loneliness														
No Threat	2.51	1.84	36	1.79	1.59	33	2.59	1.91	37	SC	1.98	2, 396	.140	.01
Implicit Threat	2.41	1.56	57	2.29	1.73	48	2.08	1.53	38	Threat	14.15	2, 396	<.001	.07
Explicit Threat	3.51	1.91	60	3.07	1.91	58	3.18	1.85	38	SC x Threat	0.85	4, 396	.496	.01
Self-esteem														
No Threat	2.15	0.72	36	2.40	0.56	33	2.25	0.82	37	SC	1.33	2, 395	.267	.01
Implicit Threat	2.21	0.67	57	2.14	0.78	47	2.34	0.70	38	Threat	1.26	2, 395	.285	.01
Explicit Threat	2.01	0.79	60	2.23	0.71	58	2.14	0.76	38	SC x Threat	0.95	4, 395	.435	.01
Body satisfaction														
No Threat	2.57	1.62	36	3.07	1.68	33	2.26	1.61	37	SC	1.63	2, 395	.196	.01
Implicit Threat	2.37	1.50	57	2.77	1.74	47	3.14	1.66	38	Threat	5.59	2, 395	.004	.03
Explicit Threat	2.03	1.76	60	2.23	1.77	58	2.14	1.68	38	SC x Threat	1.56	4, 395	.183	.02
Depression														
No Threat	0.72	0.85	36	0.34	0.56	33	0.69	0.88	37	SC	4.33	2, 395	.014	.02
Implicit Threat	0.64	0.72	57	0.64	0.80	47	0.45	0.80	38	Threat	7.76	2, 395	<.001	.04
Explicit Threat	1.15	0.92	60	0.66	0.76	58	0.92	0.89	38	SC x Threat	1.86	4, 395	.116	.02

ANOVA, analysis of variance. SC, self-complexity. *df* , degrees of freedom. MC, manipulation check.

**Table 2:** Means, standard deviations, and exploratory analyses for the interaction of the number of superaspects and social identity threat on study variables (Study 1).

Variable	No threat			Implicit threat			Explicit threat			ANOVA				
	*M*	*SD*	*n*	*M*	*SD*	*n*	*M*	*SD*	*n*	Effect	*F* ratio	*df*	*p*	$\eta_{p}^{2}$
Anger														
No Superaspect	0.82	1.20	36	1.17	1.45	60	2.73	1.67	53	Threat	21.27	2, 389	<.001	.10
One Superaspect	0.76	1.02	17	1.14	1.56	14	1.61	1.63	19	Superaspect	2.33	4, 389	.056	.02
Two Superaspects	0.71	1.12	26	0.89	1.18	29	2.63	1.72	48	Threat x Superaspect	1.44	8, 389	.176	.02
Three Superaspects	0.81	1.31	18	0.71	1.63	26	1.95	1.47	26					
≥ Four Superaspects	0.72	1.41	9	0.73	1.17	13	1.05	1.64	10					
Fear														
No Superaspect	1.67	1.71	36	1.39	1.59	60	2.08	1.62	53	Threat	0.40	2, 389	.672	.00
One Superaspect	1.58	1.66	17	1.83	1.80	14	1.72	1.77	19	Superaspect	4.81	4, 389	<.001	.05
Two Superaspects	1.10	1.43	26	1.00	1.19	29	1.48	1.43	48	Threat x Superaspect	0.49	8, 389	.863	.01
Three Superaspects	1.17	1.55	18	0.98	1.53	26	1.24	1.39	26					
≥ Four Superaspects	0.71	1.04	9	0.85	1.44	13	0.42	0.58	10					
Shame														
No Superaspect	1.93	1.84	36	1.70	1.58	60	2.90	1.81	53	Threat	4.09	2, 389	.017	.02
One Superaspect	1.71	1.77	17	1.93	1.86	14	2.13	1.94	19	Superaspect	5.40	4, 389	<.001	.05
Two Superaspects	1.35	1.52	26	1.22	1.45	29	2.62	1.82	48	Threat x Superaspect	1.06	8, 389	.390	.02
Three Superaspects	1.32	1.62	18	1.18	1.73	26	1.77	1.82	26					
≥ Four Superaspects	1.03	1.55	9	0.83	1.28	13	0.55	0.95	10					
Sense of social belonging														
No Superaspect	2.31	1.53	36	2.40	1.55	60	1.11	1.07	53	Threat	13.12	2, 389	<.001	.06
One Superaspect	2.91	1.64	17	2.39	1.53	14	2.24	1.91	19	Superaspect	6.12	4, 389	<.001	.06
Two Superaspects	2.10	1.37	26	2.51	1.15	29	0.91	1.11	48	Threat x Superaspect	1.45	8, 389	.175	.03
Three Superaspects	2.64	1.35	18	3.33	1.56	26	1.88	1.52	26					
≥ Four Superaspects	3.03	1.52	9	2.65	1.42	13	2.65	1.63	10					
Loneliness														
No Superaspect	2.63	1.72	36	2.41	1.60	61	3.92	1.67	53	Threat	3.83	2, 390	.022	.02
One Superaspect	2.78	2.07	17	2.73	1.86	14	2.66	1.94	19	Superaspect	6.45	4, 390	<.001	.06
Two Superaspects	2.09	1.73	26	2.12	1.48	29	3.42	1.93	48	Threat x Superaspect	2.04	8, 390	.041	.04
Three Superaspects	2.14	1.88	18	2.08	1.76	26	3.03	1.69	26					
≥ Four Superaspects	1.14	1.41	9	1.94	1.38	13	0.86	0.95	10					
Self-esteem														
No Superaspect	2.08	0.74	36	2.10	0.75	60	1.83	0.79	53	Threat	0.13	2, 389	.877	.00
One Superaspect	2.04	0.91	17	2.14	0.81	14	2.30	0.83	19	Superaspect	6.63	4, 389	<.001	.06
Two Superaspects	2.38	0.62	26	2.20	0.66	29	2.15	0.68	48	Threat x Superaspect	0.91	8, 389	.511	.02
Three Superaspects	2.51	0.55	18	2.46	0.64	26	2.30	0.68	26					
≥ Four Superaspects	2.55	0.51	9	2.43	0.63	13	2.75	0.25	10					
Body satisfaction														
No Superaspect	2.33	1.56	36	2.24	1.37	60	1.63	1.46	53	Threat	0.98	2, 389	.378	.00
One Superaspect	2.78	1.82	17	2.59	2.02	14	2.57	2.16	19	Superaspect	9.23	4, 389	<.001	.09
Two Superaspects	2.35	1.62	26	2.82	1.48	29	1.70	1.44	48	Threat x Superaspect	1.08	8, 389	.378	.02
Three Superaspects	3.13	1.68	18	3.47	1.91	26	2.87	1.77	26					
≥ Four Superaspects	3.24	1.66	9	3.29	1.62	13	4.10	1.40	10					
Depression														
No Superaspect	0.80	0.85	36	0.66	0.74	60	1.37	0.89	53	Threat	1.28	2, 389	.279	.01
One Superaspect	0.79	0.94	17	0.91	0.86	14	0.65	0.85	19	Superaspect	8.76	4, 389	<.001	.08
Two Superaspects	0.45	0.64	26	0.42	0.64	29	0.90	0.82	48	Threat x Superaspect	2.43	8, 389	.014	.05
Three Superaspects	0.41	0.79	18	0.59	0.96	26	0.53	0.62	26					
≥ Four Superaspects	0.19	0.33	9	0.26	0.42	13	0.03	0.09	10					

ANOVA, analysis of variance. *df* , degrees of freedom.

**Table 3:** Means, standard deviations, and analyses for the interaction of self-complexity and social identity threat on study variables (Study 2).

Variable	Low SC			High individual SC			ANOVA				
	*M*	*SD*	*n*	*M*	*SD*	*n*	Effect	*F* ratio	*df*	*p*	$\eta_{p}^{2}$
Positivity of self-aspects (MC)											
No Threat	-3.41	4.26	59	4.11	1.58	58	SC	497.07	1, 329	<.001	.60
Implicit Threat	-3.80	4.35	55	4.67	1.80	53	Threat	0.37	2, 329	.688	.00
Explicit Threat	-3.33	4.43	54	4.76	1.59	56	SC x Threat	0.59	2, 329	.553	.00
*H* (MC)											
No Threat	0.18	0.05	59	0.71	0.18	58	SC	971.07	1, 329	<.001	.75
Implicit Threat	0.18	0.06	55	0.68	0.21	53	Threat	1.15	2, 329	.317	.01
Explicit Threat	0.19	0.06	54	0.73	0.23	56	SC x Threat	0.60	2, 329	.548	.00
Social identity threat (MC)											
No Threat	1.99	1.62	59	2.06	1.39	58	SC	0.12	1, 329	.726	.00
Implicit Threat	2.86	2.06	55	2.82	1.67	53	Threat	19.42	2, 329	<.001	.11
Explicit Threat	3.31	1.61	54	3.47	1.58	56	SC x Threat	0.11	2, 329	.895	.00
Anger											
No Threat	0.68	0.98	59	0.80	1.09	58	SC	0.33	1, 329	.566	.00
Implicit Threat	1.45	1.61	55	1.39	1.39	53	Threat	61.50	2, 329	<.001	.27
Explicit Threat	2.72	1.82	54	2.94	1.63	56	SC x Threat	0.28	2, 329	.757	.00
Fear											
No Threat	1.71	1.55	59	2.00	1.41	58	SC	0.23	1, 329	.633	.00
Implicit Threat	2.36	1.90	55	1.97	1.57	53	Threat	2.09	2, 329	.125	.01
Explicit Threat	2.35	1.48	54	2.20	1.61	56	SC x Threat	1.33	2, 329	.265	.01
Shame											
No Threat	1.81	1.52	59	1.99	1.33	58	SC	0.20	1, 329	.656	.00
Implicit Threat	2.52	2.00	55	2.36	1.75	53	Threat	11.77	2, 329	<.001	.07
Explicit Threat	3.11	1.63	54	2.84	1.74	56	SC x Threat	0.54	2, 329	.582	.00
Sense of social belonging											
No Threat	2.21	1.36	59	1.77	1.19	58	SC	0.08	1, 328	.772	.00
Implicit Threat	1.85	1.55	55	2.12	1.38	53	Threat	16.80	2, 328	<.001	.09
Explicit Threat	1.09	1.07	53	1.14	1.15	56	SC x Threat	2.29	2, 328	.103	.01
Loneliness											
No Threat	2.69	1.69	59	3.19	1.25	58	SC	0.56	1, 329	.455	.00
Implicit Threat	3.05	1.69	55	2.88	1.79	53	Threat	7.39	2, 329	<.001	.04
Explicit Threat	3.63	1.54	54	3.69	1.51	56	SC x Threat	1.29	2, 329	.276	.01
Self-esteem											
No Threat	1.91	0.83	59	1.84	0.70	58	SC	0.29	1, 329	.589	.00
Implicit Threat	1.81	0.71	55	1.96	0.75	53	Threat	0.08	2, 329	.925	.00
Explicit Threat	1.82	0.68	54	1.87	0.75	56	SC x Threat	0.55	2, 329	.576	.00
Body satisfaction											
No Threat	2.44	1.57	59	2.00	1.29	58	SC	0.01	1, 329	.905	.00
Implicit Threat	2.29	1.76	55	2.46	1.54	53	Threat	1.71	2, 329	.183	.01
Explicit Threat	1.82	1.64	54	2.15	1.58	56	SC x Threat	1.89	2, 329	.152	.01
Depression											
No Threat	0.80	0.91	59	0.73	0.65	58	SC	0.47	1, 329	.494	.00
Implicit Threat	0.89	0.91	55	0.82	0.81	53	Threat	2.12	2, 329	.122	.01
Explicit Threat	1.01	0.77	54	0.96	0.83	56	SC x Threat	0.01	2, 329	.990	.00

ANOVA, analysis of variance; SC, self-complexity; *df*, degrees of freedom; MC, manipulation check.

#### Preliminary analysis

**Table 1** reports means, standard deviations, and all ANOVA omnibus effects, including manipulation checks. First, we ensured that no spillover effect might have occurred and that our experimental manipulations of self-complexity and social identity threat were successful. For significant main effects, Hochberg’s GT2 *post hoc* tests were applied to account for unequal sample sizes across conditions (46).

#### Positivity of self-aspects

We first tested whether participants in the low self-complexity condition reported a less positive self-aspect than those in the high individual and collective self-complexity conditions, based on the self-reported positivity of their self-aspects. This served two purposes: to determine whether focusing solely on the stigmatized self-aspect reduced self-aspect positivity, and to check for potential spillover effects on other self-aspects. A 3x3 ANOVA only revealed a significant main effect of the self-complexity condition, *F*(2, 392) = 174.17, *p* < .001, $\eta_{p}^{2}$ = .47. Hochberg’s GT2 *post hoc* tests revealed that the level of positivity in the low self-complexity condition (*M* = 2.62, *SD* = 2.59) significantly differed from both the high individual self-complexity condition (*M* = 6.97, *SD* = 1.72), *p* < .001, *d* = -1.96, 95%-*CI* [-1.68; -2.24], and the high collective self-complexity condition (*M* = 6.79, *SD* = 2.07), *p* < .001, *d* = -1.75, 95%-*CI* [-1.46; -2.04]. These results indicate that a sole, stigmatized self-aspect is associated with lower self-concept positivity, and that no spillover effect occurred.

#### Manipulation checks

First, we tested whether the manipulation of self-complexity was successful, using the *H*-score. A 3x3 factorial ANOVA only revealed a significant main effect of self-complexity, *F*(2, 396) = 147.78, *p* < .001, $\eta_{p}^{2}$ = .43. Hochberg’s GT2 *post hoc* tests revealed that participants in the control condition had a significant less complex self-concept (*M* = 0.22, *SD* = 0.08) than participants in both the high individual self-complexity (*M* = 0.58, *SD* = 0.26), *p* < .001, *d* = -1.91, 95%-*CI* [-2.19; -1.63], and the high collective self-complexity conditions (*M* = 0.55, *SD* = 0.19), *p* < .001, *d* = -2.39, 95%-*CI* [-2.71; -2.08]. In sum, we concluded that the experimental manipulation of self-complexity was successful.

Next, we tested whether our social identity threat manipulation was successful. A 3x3 factorial ANOVA only revealed a significant main effect of social identity threat, *F*(2, 396) = 15.95, *p* < .001, $\eta_{p}^{2}$ = .07. Hochberg’s GT2 *post hoc* tests revealed that measured social identity threat was significantly higher in the explicit threat condition (*M* = 2.95, *SD* = 1.88) than in both the implicit threat (*M* = 1.98, *SD* = 1.69), *p* < .001, *d* = 0.54, 95%-*CI* [0.31; 0.77], and the control conditions (*M* = 1.95, *SD* = 1.79), *p* < .001, *d* = 0.54, 95%-*CI* [0.29; 0.79]. These results suggest that only participants in the explicit threat condition experienced social identity threat, but not participants in the implicit threat and no threat control conditions. In sum, we concluded that the experimental manipulation of social identity threat was partially successful.

#### Hypothesis testing

##### Affective responses

For anger, we only found a significant main effect of social identity threat, *F*(2, 395) = 44.85, *p* < .001, $\eta_{p}^{2}$ = .19. Hochberg’s GT2 *post hoc* tests revealed that participants in the explicit threat condition felt angrier (*M* = 2.33, *SD* = 1.71) than participants in both the implicit threat (*M* = 0.99, *SD* = 1.42), *p* < .001, *d* = 0.85, 95%-*CI* [0.61; 1.09], and the control conditions (*M* = 0.77, *SD* = 1.17), *p* < .001, *d* = 1.03, 95%-*CI* [0.77; 1.29].

For shame, we only found a significant main effect of social identity threat, *F*(2, 395) = 12.66, *p* < .001, $\eta_{p}^{2}$ = .06. Hochberg’s GT2 *post hoc* tests revealed that participants in the explicit threat condition felt more shame (*M* = 2.38, *SD* = 1.88) than participants in both the implicit threat (*M* = 1.45, *SD* = 1.60), *p* < .001, *d* = 0.53, 95%-*CI* [0.30; 0.76], and the control conditions (*M* = 1.57, *SD* = 1.69), *p* < .001, *d* = 0.45, 95%-*CI* [0.20; 0.70]. Taken together, these results did not support our hypothesis.

##### Sense of social belonging

For sense of social belonging, we only found a significant main effect of social identity threat, *F*(2, 395) = 29.55, *p* < .001, $\eta_{p}^{2}$ = .13. Hochberg’s GT2 *post hoc* tests revealed that sense of social belonging was significantly lower in the explicit threat condition (*M* = 1.41, *SD* = 1.43) than in both the implicit threat (*M* = 2.61, *SD* = 1.49), *p* < .001, *d* = -0.82, 95%-*CI* [-1.06; -0.59], and the control conditions (*M* = 2.47, *SD* = 1.49), *p* < .001, *d* = -0.73, 95%-*CI* [-0.98; -0.47]. These results did not support our hypothesis.

##### Loneliness

For loneliness, we only found a significant main effect of social identity threat, *F*(2, 396) = 14.15, *p* < .001, $\eta_{p}^{2}$ = .07. Hochberg’s GT2 *post hoc* tests revealed that feelings of loneliness were significantly higher in the explicit threat condition (*M* = 3.27, *SD* = 1.90) than in both the implicit threat (*M* = 2.28, *SD* = 1.61), *p* < .001, *d* = 0.56, 95%-*CI* [0.32; 0.79], and the control conditions (*M* = 2.31, *SD* = 1.81), *p* < .001, *d* = 0.51, 95%-*CI* [0.26; 0.77]. These results did not support our hypothesis.

##### Body satisfaction

For body satisfaction, we only found a significant main effect of social identity threat, *F*(2, 395) = 5.59, *p* = .004, $\eta_{p}^{2}$ = .03. Hochberg’s GT2 *post hoc* tests revealed that body satisfaction was significantly lower in the explicit threat condition (*M* = 2.13, *SD* = 1.73) than in the implicit threat condition (*M* = 2.71, *SD* = 1.64), *p* = .009, *d* = -0.34, 95%-*CI* [-0.57; -0.11]. These results did not support our hypothesis.

##### Depression

For depression, we found a significant main effect of self-complexity, *F*(2, 395) = 4.33, *p* = .014, $\eta_{p}^{2}$ = .02. Hochberg’s GT2 *post hoc* tests revealed that depressive symptoms were significantly lower in the high individual self-complexity condition (*M* = 0.58, *SD* = 0.74) than in the low self-complexity condition (*M* = 0.86, *SD* = 0.86), *p* = .010, *d* = -0.35, 95%-*CI* [-0.58; -0.12]. In addition, we found a significant main effect of social identity threat, *F*(2, 395) = 7.76, *p* < .001, $\eta_{p}^{2}$ = .04. Hochberg’s GT2 *post hoc* tests revealed that depressive symptoms were significantly higher in the explicit threat condition than (*M* = 0.91, *SD* = 0.88) in both the implicit threat (*M* = 0.59, *SD* = 0.77), *p* = .002, *d* = 0.39, 95%-*CI* [0.16; 0.61], and the control conditions (*M* = 0.59, *SD* = 0.79), *p* = .006, *d* = 0.38, 95%-*CI* [0.13; 0.63]. These results supported our antagonistic main effect hypothesis.

##### Interim conclusion

In sum, we did not find support for the buffering hypothesis (i.e., in terms of an interaction effect) of high individual and collective self-complexity on the negative consequences of weight-based social identity threat. However, for depression, we found an independent main effect of self-complexity, indicating that a high individual self-complexity can lower depressive symptoms. In addition, we found that exposure to explicit weight-based social identity threat led to stronger feelings of anger and shame, a lower sense of social belonging, increased feelings of loneliness, lower body satisfaction, and higher levels of depressive symptoms. However, we could not successfully manipulate implicit weight-based social identity threat and thus could not observe any corresponding effects.

#### Exploratory analysis

Although not initially theorized in the self-complexity model (26, 27), research suggests that the quality of self-aspects may play a role in buffering negative effects on well-being. Superaspects, defined as self-aspects rated above the midpoint on a positivity scale, may be particularly effective in buffering negative experiences because their strong positive valence makes them especially potent sources of self-evaluation and may shield individuals from severe negative self-beliefs (e.g., 28, 30, 34, 47). When encountered in a stressful situation, superaspects should more strongly pull momentary self-evaluation in a favorable direction, thereby creating greater evaluative contrast, attenuating the relative weight of the activated negative self-aspect, reducing affective spillover, and restoring self-integrity more effectively than ordinary positive self-aspects (28, 34, 47).

To test the main and buffering effects of superaspects, we counted the number of superaspects per participant, grouped them into five categories (with four or more as the highest), and conducted a series of 5x3 ANOVAs. Full results are reported in **Table 2**. Means and standard deviations for the main effects are reported in the **Supplementary Material**. Overall, the analyses revealed clear antagonistic main effects of weight-based social identity threat and the superaspect count on well-being outcomes. Social identity threat showed significant main effects on anger, shame, and sense of social belonging. Hochberg’s GT2 *post hoc* comparisons indicated that participants in the explicit threat condition reported significantly higher anger and shame and a significantly lower sense of social belonging than participants in both the implicit threat and the control conditions (*M*s_Diff_ ≥ |0.81|, *SE*s ≤ 0.21, 95%-*CI*s [≥ |0.30|; ≤ |1.99|], *p*s ≤ .001).

In contrast, the superaspect count demonstrated significant main effects on fear, shame, sense of social belonging, self-esteem, and body satisfaction, indicating a consistent beneficial effect of higher self-complexity. Hochberg’s GT2 *post hoc* comparisons showed that participants with three superaspects reported significantly less shame, higher levels of social belonging, higher self-esteem and higher body satisfaction compared to participants with no superaspects (*M*s_Diff_ ≥ |0.41|, *SE*s ≤ 0.25, 95%-*CI*s [≥ |0.05|; ≤ |1.77|], *p*s ≤ .025). Similarly, participants with four or more superaspects consistently reported more favorable outcomes across all dependent variables compared to those with no superaspects, including less fear and shame as well as higher social belonging, self-esteem and body satisfaction (*M*s_Diff_ ≥ |0.56|, *SE*s ≤ 0.33, 95%-*CI*s [≥ |0.06|; ≤ |2.37|], *p*s ≤ .024).

In addition, significant interaction effects emerged for loneliness, *F*(8, 390) = 2.04, *p* = .041, $\eta_{p}^{2}$ = .04, and depression, *F*(8, 389) = 2.43, *p* = .014, $\eta_{p}^{2}$ = .05. For loneliness, simple main effects showed that within the explicit threat condition, participants with no superaspects reported significantly higher loneliness than participants with one, three, and four or more superaspects (*Ms_Diff_* ≥ 0.89, *SEs* ≤ 0.59, 95%-*CIs* [0.08; 4.22], *ps* ≤ .032). Moreover, participants with one, two, and three superaspects reported significantly higher loneliness than those with four or more superaspects (*M*s_Diff_ ≥ 1.80, *SEs* ≤ 0.67, 95%-*CIs* [0.48; 3.73], *ps* ≤ .008). Overall, these results indicate that a higher number of superaspects reduced loneliness under explicit threat.

A similar pattern emerged for depression. Within the explicit threat condition, participants with no superaspects reported significantly higher depression than participants with one, two, three, and four or more superaspects (*M*s_Diff_ ≥ 0.47, *SEs* ≤ 0.27, 95%-*CIs* [0.16; 1.87], *ps* ≤ .003). Participants with two superaspects reported significantly higher depression than participants with three superaspects (*M*_Diff_ = 0.37, *SE* ≤ 0.19, 95%-*CIs* [0.00; 0.75], *p* = .050). In addition, depression levels were significantly higher among participants with one or two superaspects compared to those with four or more superaspects (*M*s_Diff_ ≥ 0.62, *SEs* ≤ 0.30, 95%-*CIs* [0.03; 1.41], *ps* ≤ .041). Taken together, these findings suggest that higher self-complexity counteracted the adverse effects of explicit weight-based social identity threat on loneliness and depression.

### Discussion

Study 1 examined whether larger-bodied people might benefit from high individual or collective self-complexity to counteract the negative effects of weight-based social identity threat. The results of our hypothesis tests did not provide support for the buffering effect of high individual and collective self-complexity on the negative consequences of weight-based social identity threat. However, we found an antagonistic main effect of high individual self-complexity on depression, which suggests that high individual self-complexity decreases depressive symptoms independently of the experience of weight-based social identity threat. Moreover, we did not find a buffering effect of high collective self-complexity. This finding contradicts previous findings of the social cure literature (e.g., 28, 30, 32), indicating that larger-bodied people do not benefit from group memberships. For larger-bodied people, high collective self-complexity might not provide a sense of connectedness or social and psychological resources associated with their group memberships. Taken together, the results indicate that if a buffering effect occurs, it is due to high individual self-complexity.

Exploratory analyses showed that reporting more superaspects had a positive main effect on participants’ well-being. Participants with a larger number of superaspects reported less fear and shame, a greater sense of social belonging, higher levels of self-esteem and higher levels of body satisfaction. In addition, we found two interaction effects, indicating a buffering effect of more superaspects on the negative effects of weight-based social identity threat on loneliness and depression for participants in the explicit threat condition. In sum, these results could suggest that the positivity of self-aspects enables the buffering effect of self-complexity, which is in line with prior research (e.g., 34, 47–49). These results and interpretations, based on exploratory analyses and plenty of *post hoc* comparisons, should be viewed as preliminary rather than conclusive. The former limitation is in need of a further experiment with profound a-priori hypotheses, the latter limitation can be addressed by considering the family-wise error rate. Applying appropriate methods, e.g., Bonferroni–Holm, the observed pattern of evidence remained rather stable (see especially *p*s <.001 in **Table 2**).

Given these insights, we refined our research design in Study 2 to further investigate the factors that contribute to the antagonistic main and buffering effects of self-complexity. First, we streamlined the experimental design by focusing only on the comparison between low and high individual self-complexity, as high collective self-complexity did not show a significant influence in Study 1. Second, we modified the instructions for the high self-complexity condition, asking participants to list only important positive self-aspects. This change was made to account for the finding of Study 1 that superaspects are responsible for the antagonistic main and buffering effects of high self-complexity. Third, we refined the social identity threat manipulation text because the manipulation check indicated that the implicit threat manipulation did not elicit social identity threat.

## Study 2

Building on the findings from Study 1, Study 2 was designed to further investigate the factors that might contribute to the antagonistic main and/or buffering effect of self-complexity. More precisely, we focused on the effects of a high positive individual self-complexity and weight-based social identity threat on well-being. For the well-being measures, our hypotheses remained unchanged from Study 1.

### Method

#### Participants

An a-priori power analysis for a 2x3 ANOVA using G*Power (36) revealed that we needed to collect a minimum of *N* = 318 to detect a small to medium effect of *f* = 0.175, assuming an *α* = .05 and *1-β* = .80. Participants were recruited via an online sampling provider (*Prolific*; www.prolific.com). Study 2 was introduced to participants as an investigation of how one is perceived by others in social interactions. We decided to oversample by approximately 10% to account for potential drop-out. To ensure equal sample sizes across experimental conditions, we recruited 354 larger-bodied people living in Germany. Nineteen participants were excluded from the analysis due to double participation, failed attention checks, or not following task instructions of the self-complexity task. This resulted in a total sample size of 335 participants. Based on self-reported BMI, 182 participants (54.33%) had a BMI between 25.0 and 29.9, and 153 participants (45.67%) had a BMI of 30.0 or higher (no missing values). The average age of the sample was *M* = 33.32 years (*SD* = 9.67). One hundred and eighty-one (54.03%) participants were male, 146 (43.58%) participants were female, and seven (2.09%) participants reported to be non-binary (1 missing value), 112 (33.43%) participants reported having a migration background (no missing values), 190 (56.72%) participants had a college or university degree (two missing values), and 40 (11.94%) participants reported to be a supporter of the body positivity movement (29 missing values).

#### Design and procedure

We conducted an online experiment with a 2x3 factorial between-subjects design manipulating individual self-complexity (low vs. high) and the salience of social identity threat (no vs. implicit vs. explicit), with the same dependent variables as in Study 1. Study 2 used an analogous procedure to Study 1, with some adjustments to both experimental manipulations. First, the self-complexity manipulation was refined to emphasize the inclusion of positive and personally meaningful self-aspects. In the high self-complexity condition, participants ascribed at least two attributes to their self-aspect “I am a larger-bodied person” and rated its positivity, before listing three additional important positive self-aspects that describe them in a personal and individual way. Below the instructions, free text lines were provided for their responses. After that, participants rated the positivity of the three self-aspects. In the low self-complexity condition, participants only ascribed at least two attributes to the self-aspect “I am a larger-bodied person,” followed by the positivity rating of this self-aspect.

Second, the social identity threat manipulation was revised based on Study 1, which indicated that the implicit threat condition was ineffective. In the implicit threat condition, participants now read that some people at the neighboring table were having a conversation about eating habits and dietary advice, instead of receiving no information about the conversation topic. Thus, the manipulation now entailed multiple cues relevant to the self-aspect of being larger-bodied (i.e., a potentially evaluative gaze, a potentially unpleasant conversation topic, and a stereotypical eating context), which we expected to increase the salience of social identity threat compared to Study 1. This change was also included in the explicit threat condition to keep the conditions parallel, while the clearly negative comment about the eating behavior of the participant still defined the line to an explicit threat. In the no threat control condition, participants read that some people at the neighboring table on their right side were having a conversation about their indoor plants and which varieties thrive best in each room.

#### Materials

Materials were the same as in Study 1, except for the positivity of each self-aspect. Participants rated each self-aspect on a 20-point scale ranging from -10 (very negative) to 10 (very positive). All scales showed good to very good internal consistencies. Cronbach’s alpha (α) was .98 for social identity threat, .95 for anger, .93 for fear, .91 for shame, .93 for sense of social belonging, .95 for loneliness, .92 for self-esteem, .96 for body satisfaction, and .93 for depression.

### Results

#### Preliminary analysis

**Table 3** reports means, standard deviations, and all ANOVA omnibus effects, including manipulation checks. In case of a significant main effect, we performed Games-Howell *post hoc* tests to account for violations against variance homogeneity (46). The test is based on Welch’s degrees of freedom correction.

#### Manipulation checks

To test whether our experimental manipulations of self-complexity and social identity threat were successful, we performed three 2x3 factorial ANOVAs. As seen in **Table 3**, both manipulations were successful.

For self-complexity, we tested whether the positivity of self-aspects significantly differed between low and high self-complexity conditions and whether participants differed in their level of self-complexity between both conditions. First, a 2x3 factorial ANOVA on positivity of self-aspects only revealed a significant main effect of self-complexity, *F*(1, 329) = 497.07, *p* < .001, $\eta_{p}^{2}$ = .60. Specifically, positivity of self-aspects was significantly lower in the low self-complexity condition (*M* = -3.51, *SD* = 4.33) than in the high individual self-complexity condition (*M* = 4.51, *SD* = 1.67), indicating that the positivity instruction worked and no spillover effect occurred in the high self-complexity condition. Second, the *H*-score was significantly lower in the low self-complexity condition (*M* = 0.19, *SD* = 0.06) compared to the high self-complexity condition (*M* = 0.71, *SD* = 0.21), *F*(1, 329) = 971.07, *p* < .001, $\eta_{p}^{2}$ = .75, indicating that participants in the high individual self-complexity condition had a more complex self-concept.

For social identity threat, a 2x3 factorial ANOVA revealed a significant main effect of the social identity threat manipulation, *F*(2, 329) = 19.42, *p* < .001, $\eta_{p}^{2}$ = .11. Games-Howell *post hoc* tests revealed that measured social identity threat was significantly higher in the explicit threat condition (*M* = 3.39, *SD* = 1.59) compared to the control condition (*M* = 2.03, *SD* = 1.51), *t*(221.94) = 6.65, *p* < .001, *d* = 0.88, 95%-*CI* [0.60; 1.15]. Furthermore, measured social identity threat was significantly higher in the implicit threat condition (*M* = 2.84, *SD* = 1.87) compared to the control condition, *t*(205.64) = 3.59, *p* = .001, *d* = 0.48, 95%-*CI* [0.22; 0.74]. These results indicate that participants in the explicit and implicit threat conditions experienced higher levels of social identity threat compared to participants in the no threat control condition. Furthermore, in contrast to Study 1, we can conclude that the refined and enhanced implicit threat condition was successful in inducing social identity threat. Since the levels of induced threat in the implicit and explicit threat conditions are descriptively different in the expected direction but marginally non-significant and at the same time show a medium effect size, *t*(209.47) = 2.35, *p* = .051, *d* = .32, 95%-*CI* [0.05; 0.58], the distinction between those levels is rather ambiguous. Nevertheless, it is unambiguous enough to proceed with hypothesis testing.

#### Hypothesis testing

As seen in **Table 3**, we only found significant main effects of the social identity threat manipulation for shame, anger, sense of social belonging, and loneliness. Taken together, the results did not provide evidence for a buffering effect of a high positive self-complexity on the negative consequences of weight-based social identity threat.

#### Affective responses

For anger, Games-Howell *post hoc* tests revealed that participants felt angrier in the explicit threat condition (*M* = 2.83, *SD* = 1.72) than in both the implicit threat (*M* = 1.42, *SD* = 1.50), *t*(212.83) = 6.47, *p* < .001, *d* = 0.87, 95%-*CI* [0.59; 1.15] and the control conditions (*M* = 0.74, *SD* = 1.03), *t*(176.06) = 11.02, *p* < .001, *d* = 1.49, 95%-*CI* [1.19; 1.78]. Furthermore, participants felt angrier in the implicit threat condition than in the control condition, *t*(188.11) = 3.94, *p* < .001, *d* = 0.53, 95%-*CI* [0.27; 0.80].

For shame, Games-Howell *post hoc* tests revealed that participants felt more shame in the explicit threat condition (*M* = 2.97, *SD* = 1.69) than did participants in the control condition (*M* = 1.90, *SD* = 1.43), *t*(214.14) = 5.17, *p* < .001, *d* = 0.69, 95%-*CI* [0.42; 0.95]. Furthermore, participants felt more shame in the implicit threat condition (*M* = 2.44, *SD* = 1.88) than in the control condition, *t*(199.47) = 2.43, *p* = .043, *d* = 0.33, 95%-*CI* [0.06; 0.59].

##### Sense of social belonging

Games-Howell *post hoc* tests revealed that sense of social belonging was significantly lower in the explicit threat condition (*M* = 1.11, *SD* = 1.11) than in both the implicit threat (*M* = 1.98, *SD* = 1.47), *t*(198.93) = 4.93, *p* < .001, *d* = -0.67, 95%-*CI* [-0.94; -0.39] and the control conditions (*M* = 1.99, *SD* = 1.29), *t*(222.48) = 5.50, *p* < .001, *d* = -0.73, 95%-*CI* [-1.00; -0.46].

##### Loneliness

Games-Howell *post hoc* tests revealed that loneliness was significantly higher in the explicit threat condition (*M* = 3.66, *SD* = 1.52) compared to both in the implicit threat (*M* = 2.97, *SD* = 1.73), *t*(211.29) = 3.14, *p* = .005, *d* = 0.42, 95%-*CI* [0.16; 0.69], and the control conditions (*M* = 2.94, *SD* = 1.50), *t*(223.85) = 3.61, *p* = .001, *d* = 0.48, 95%-*CI* [0.21; 0.74].

### Discussion

Building on the findings of Study 1, Study 2 examined whether a high positive individual self-complexity exerts an antagonistic main effect on, or interaction effects buffering the negative effects of weight-based social identity threat. To this end, we adjusted our research design, addressing the retrospective imperfections of Study 1. Preliminary analyses and the manipulation checks confirmed that our adjustment and enhancement resulted in successfully inducing social identity threat. While the distinction between the no-threat and implicit threat conditions became unambiguous, the distinction between the implicit and explicit threat conditions became slightly ambiguous. Therefore, differences between the implicit and explicit threat conditions should be interpreted with appropriate caution. Despite these improvements, the results of Study 2 did not support our hypotheses. High positive individual self-complexity did not buffer the negative effects of weight-based social identity threat, nor did it show an antagonistic main effect on any outcome variable. These findings challenge the theoretical assumption that a more complex positive self-concept inherently offers resilience against negative stressors such as social identity threat (e.g., 28, 30, 34, 47). A possible explanation for the lack of effects could lie in the revised manipulation. Participants in the high self-complexity condition were instructed to generate exactly three personally important and positive self-aspects. This restriction in quantity and quality may have limited the extent to which participants could depict their natural self-concept and its natural complexity (see 26, 27). In turn, the antagonistic main and buffering effects may also have been limited. Another possibility is that merely generating self-aspects may not always be sufficient; incorporating an element of affirmation—such as writing about their importance—might be necessary to reliably elicit an effect.

Study 2 replicated the direct negative effects of weight-based social identity threat across various measures, including anger, shame, sense of social belonging, and loneliness. These results highlight the robust influence of social identity threat on well-being (25). Explicit weight-based social identity threat appears especially harmful, as it increased negative emotional responses and loneliness while reducing sense of social belonging. However, Study 2 did not replicate the main effects of weight-based social identity threat on body satisfaction and depression. This might be due to several reasons discussed in the General Discussion, especially given the limited robustness and generalizability of the antagonistic main and buffering hypotheses.

## General discussion

This article examined whether high self-complexity positively impacts well-being among larger-bodied people and thus counteracts the negative effect of weight-based social identity threat. The results of both studies provided limited evidence for the antagonistic main effects. The results of both studies provided limited evidence for the antagonistic main effects. Study 1 indicated that antagonistic main or buffering effects can occur if participants are high in individual self-complexity and have at least three to four superaspects (28). This finding is in line with the theoretical reasoning that a more positive complex self-concept can provide psychological resources to counteract a negative stressor (34, 47–49). Study 2 did not replicate these findings, raising questions about the robustness and generalizability of the results. Replication of the exploratory analyses of Study 1, with preregistered hypotheses and a parsimonious design, dissolving the need for false discovery rate adjustments, is recommended before drawing firm and sturdy theoretical or practical implications.

### Theoretical considerations and implications

Several theoretical explanations may account for the absence of a protective effect of high self-complexity. First, possessing a stigmatized self-aspect may render it highly central to one’s self-definition, especially in situations entailing an implicit or explicit threat, overshadowing other self-aspects and dominating momentary self-evaluation rather than being mitigated by them (50). As a result, otherwise valued self-aspects may be perceived as less relevant for one’s self-definition in a given threatful situation, despite their importance for general well-being. Second, possessing a stigmatized self-aspect may be the source for internal conflict due to a lack of compatibility between self-aspects (50, 51). For example, perceiving oneself as larger-bodied but also active and healthy. When self-aspects are perceived as incompatible, their simultaneous activation may not provide additive psychological resources but instead create tension within the overall self-concept. Third, the beneficial effects of self-complexity may depend on how individuals cognitively engage with stigmatizing situations (52). Translating available positive self-aspects (i.e., superaspects) into a protective response may require actively affirming these aspects in a threatful situation (53). From this perspective, self-complexity provides the necessary psychological resources, but their effectiveness may depend on whether individuals are able to draw on them in a way that supports a more adaptive appraisal of the situation. Fourth, positive effects of high self-complexity may not fully unfold immediately but become more evident in post-event recovery, when individuals can more readily shift attention toward positive self-aspects and restore self-integrity. Our state measures capturing well-being in the threatful situation may have missed the middle- and long-term effects of self-complexity. Accordingly, individuals high in self-complexity may be better able to switch to positive self-aspects more quickly and effectively following such situations.

Together, these considerations suggest that the effectiveness of self-complexity, at least its short-term effectiveness, is limited under conditions in which a stigmatized self-aspect remains highly central and incompatible with other aspects of the self. This raises the question for future research of how important self-aspects or superaspects can be effectively strengthened and utilized in stigmatizing situations to mitigate their negative consequences. It also highlights the need for a multimethodological approach to examine middle- and long-term effects of self-complexity but also the causal effects.

### Methodological and sample-related considerations

Potential explanations for the different results between both studies might be due to the refined manipulation of self-complexity in Study 2 and the different demographics of the participants in Study 1 and 2. Although Study 2 successfully manipulated low versus high individual self-complexity, it is possible that generating exactly three meaningful and positive self-aspects was insufficient to fully reflect participants’ natural self-concept, thereby limiting its capacity to counteract or buffer against weight-based social identity threat (26, 27). Instead, a larger number of positive self-aspects may be needed to counteract the detrimental health effects of weight-based social identity threat.

Demographic differences between the samples in Studies 1 and 2 may also explain the divergent findings. Compared to Study 1, Study 2’s sample was younger, included fewer participants in higher BMI ranges (BMI ≥ 30), included more people with a migration background, and was more highly educated. First, research suggests that older adults tend to have a more compartmentalized and robust self-concept, with separate categories for positive and negative self-aspects, which is shaped by diverse life experiences, social roles, and achievements (26, 27, 54). In contrast, younger people may still be in process of forming their self-concept, making them more vulnerable to the negative effects of stress (54). Second, the difference in body weight might be especially important because the visibility of having a larger body can result in more intense experiences of social identity threat (19). Thus, interventions targeting self-complexity might be more relevant and effective for individuals in higher weight groups. Third, cultural perspectives on weight and body image may influence how people perceive and internalize weight stigma (2). People with a migration background may be less exposed to, or less affected by, Western thin-ideal norms, reducing their vulnerability to social identity threat and the need to employ a coping strategy (2). Finally, higher education was found to be a resilience factor because highly educated people might possess more effective coping strategies or seeking professional help (55). Thus, participants in Study 2 might have possessed better tools to deal with weight-based social identity threat, reducing the additional buffering impact that high self-complexity might have.

### Strengths of the present research

Despite the mixed results of our studies, the present research has also some notable strengths. First, to our knowledge this research is the first to test the positive and buffering effects of high self-complexity on well-being among a highly stigmatized group. Second, the results of both studies demonstrate that the mental imagery task is a reliable method to induce explicit social identity threat and explore its consequences (25). Third, the results supported the robustness of the negative effects of explicit weight-based social identity threat on well-being. In particular, it increased negative affective reactions (i.e., anger and shame), decreased sense of social belonging, and increased feelings of loneliness. Therefore, our results with two samples of larger-bodied people corroborate those of Loreth and Simon (25), thereby enhancing the generalizability of their findings.

### Implications for future research

The results reported in this article point to several directions for future research. First, future research should further explore the conditions under which self-complexity might operate as an antagonist or buffer. The findings of Study 1 suggest that the positivity of self-aspects might play an important role in counteracting the negative effects of social identity threat. Therefore, future research should explore whether a larger number of superaspects are necessary to unlock positive effects. Future research should also refine and test different experimental manipulations of self-complexity to ensure their effectiveness and robustness. For example, to test whether an element of affirmation might be necessary to reliably elicit a positive effect. Second, future research should also address the absence of the positive effect of high collective self-complexity on well-being in Study 1. It might be possible that listing group affiliations and their associated attributes was not sufficient to activate the psychological resources typically associated with high collective self-complexity. Therefore, future studies should consider alternative ways to operationalize and measure high collective self-complexity, such as elaborating on the qualities of one’s group memberships and how those group memberships can help (e.g., 32). It may also be necessary to account for middle- and long-term effects. Third, the potential moderating role of demographic variables, such as age, migration background, level of education, and body weight should be addressed. This would help to determine whether the positive effect of self-complexity is consistent across different populations.

## Conclusion

Across two experiments, the present research provides nuanced insights into the role of self-complexity in the context of weight-based social identity threat. While it does not provide consistent evidence that high self-complexity reliably protects larger-bodied people from the detrimental effects of weight-based social identity threat, exploratory findings point to the potential relevance of highly positive self-aspects (i.e., superaspects) as a promising avenue for resilience. At the same time, the findings highlight the robust and consistent impact of explicit weight-based social identity threat on well-being, underscoring the importance of better understanding how individuals can be supported in such contexts and, even more fundamental, how these contexts can be minimized through societal awareness and acceptance. Taken together, these results refine existing assumptions about the self-complexity framework by identifying important boundary conditions and emphasizing that not all forms of self-complexity may be equally effective. Importantly, the present findings open new directions for future research, particularly in identifying when and how positive self-aspects can be more effectively activated to counteract the negative consequences of social identity threat.

## Data Availability

The raw data supporting the conclusions of this article will be made available by the authors, without undue reservation.

## Footnotes

1 We acknowledge that BMI is an imperfect proxy for health and embodiment. For example, it conflates adiposity with muscularity and was normed primarily on white, male populations, limiting validity across ethnic and gender groups (37). To reduce the risk of reifying stigma, we therefore paired the numeric cutoff with participants’ self-categorization as “larger-bodied,” ensuring that subjective identity and lived experience complement the survey contents. Nevertheless, an additional measurement of and reliance on BMI aligns our sample with prior research samples (e.g., 18).
