# Supplementary material for: Independent and opposing effects of self-complexity and weight-based social identity threat on well-being among larger-bodied people
Source: Front Psychiatry. 2026 Jun 10;17:1810126. doi: 10.3389/fpsyt.2026.1810126 (PMC13290866; doi:10.3389/fpsyt.2026.1810126)
Supplement: Supplementary file 1 [file Table1.docx]

**Supplementary Material**

*Means and standard deviations for the main effects of the number of superaspects and social identity threat on study variables (Study 1)*

| Variable | Anger | | | Fear | | | Shame | | | Sense of social belonging | | |
| --- | --- | --- | --- | --- | --- | --- | --- | --- | --- | --- | --- | --- |
|  | *M* | *SD* | *n* | *M* | *SD* | *n* | *M* | *SD* | *n* | *M* | *SD* | *n* |
| Number of superaspects |  |  |  |  |  |  |  |  |  |  |  |  |
| No Superaspect | 1.64 | 1.68 | 149 | 1.70 | 1.65 | 149 | 2.18 | 1.80 | 149 | 1.92 | 1.51 | 149 |
| One Superaspect | 1.19 | 1.45 | 50 | 1.70 | 1.71 | 50 | 1.93 | 1.83 | 50 | 2.51 | 1.71 | 50 |
| Two Superaspects | 1.66 | 1.70 | 103 | 1.25 | 1.37 | 103 | 1.90 | 1.76 | 103 | 1.66 | 1.38 | 103 |
| Three Superaspects | 1.20 | 1.58 | 70 | 1.13 | 1.47 | 70 | 1.44 | 1.73 | 70 | 2.61 | 1.60 | 70 |
| ≥ Four Superaspects | 0.83 | 1.36 | 32 | 0.68 | 1.10 | 32 | 0.80 | 1.25 | 32 | 2.76 | 1.48 | 32 |
| Social idenity threat |  |  |  |  |  |  |  |  |  |  |  |  |
| No Threat | 0.77 | 1.17 | 106 | 1.35 | 1.57 | 106 | 1.57 | 1.69 | 106 | 2.47 | 1.49 | 106 |
| Implicit Threat | 0.99 | 1.42 | 142 | 1.23 | 1.52 | 142 | 1.45 | 1.60 | 142 | 2.61 | 1.49 | 142 |
| Explicit Threat | 2.33 | 1.71 | 156 | 1.60 | 1.54 | 156 | 2.38 | 1.88 | 156 | 1.41 | 1.43 | 156 |
|  |  |  |  |  |  |  |  |  |  |  |  |  |
|  | Loneliness | | | Self-esteem | | | Body satisfaction | | | Depression | | |
|  | *M* | *SD* | *n* | *M* | *SD* | *n* | *M* | *SD* | *n* | *M* | *SD* | *n* |
| Number of superaspects |  |  |  |  |  |  |  |  |  |  |  |  |
| No Superaspect | 2.99 | 1.78 | 150 | 2.00 | 0.77 | 149 | 2.04 | 1.47 | 149 | 0.94 | 0.88 | 149 |
| One Superaspect | 2.72 | 1.93 | 50 | 2.16 | 0.84 | 50 | 2.65 | 1.97 | 50 | 0.77 | 0.88 | 50 |
| Two Superaspects | 2.72 | 1.86 | 103 | 2.22 | 0.66 | 103 | 2.18 | 1.56 | 103 | 0.65 | 0.76 | 103 |
| Three Superaspects | 2.45 | 1.80 | 70 | 2.41 | 0.63 | 70 | 3.16 | 1.79 | 70 | 0.52 | 0.80 | 70 |
| ≥ Four Superaspects | 1.38 | 1.32 | 32 | 2.56 | 0.51 | 32 | 3.53 | 1.57 | 32 | 0.17 | 0.33 | 32 |
| Social idenity threat |  |  |  |  |  |  |  |  |  |  |  |  |
| No Threat | 2.31 | 1.81 | 106 | 2.26 | 0.71 | 106 | 2.62 | 1.65 | 106 | 0.59 | 0.79 | 106 |
| Implicit Threat | 2.28 | 1.61 | 143 | 2.22 | 0.72 | 142 | 2.71 | 1.64 | 142 | 0.59 | 0.77 | 142 |
| Explicit Threat | 3.27 | 1.90 | 156 | 2.12 | 0.76 | 156 | 2.13 | 1.73 | 156 | 0.91 | 0.88 | 156 |
